# A Feasibility Study of Geometric-Decomposition Coil Compression in MRI Radial Acquisitions

**DOI:** 10.1155/2017/7685208

**Published:** 2017-06-04

**Authors:** Jing Wang, Zhifeng Chen, Yiran Wang, Lixia Yuan, Ling Xia

**Affiliations:** ^1^Department of Biomedical Engineering, Zhejiang University, Hangzhou 310027, China; ^2^Center for Brain Imaging Science and Technology, Department of Biomedical Engineering, Zhejiang University, Hangzhou, China; ^3^State Key Lab of CAD&CG, Zhejiang University, Hangzhou, China

## Abstract

Receiver arrays with a large number of coil elements are becoming progressively available because of their increased signal-to-noise ratio (SNR) and enhanced parallel imaging performance. However, longer reconstruction time and intensive computational cost have become significant concerns as the number of channels increases, especially in some iterative reconstructions. Coil compression can effectively solve this problem by linearly combining the raw data from multiple coils into fewer virtual coils. In this work, geometric-decomposition coil compression (GCC) is applied to radial sampling (both linear-angle and golden-angle patterns are discussed) for better compression. GCC, which is different from directly compressing in *k*-space, is performed separately in each spatial location along the fully sampled directions, then followed by an additional alignment step to guarantee the smoothness of the virtual coil sensitivities. Both numerical simulation data and in vivo data were tested. Experimental results demonstrated that the GCC algorithm can achieve higher SNR and lower normalized root mean squared error values than the conventional principal component analysis approach in radial acquisitions.

## 1. Introduction

In the past two decades, with the introduction of multichannel receivers, parallel imaging (PI) [1, 2] has experienced rapid advance from basic technological development to a wide range of clinical applications. This has significantly accelerated data acquisitions in magnetic resonance imaging (MRI) and led research to apply large coil arrays with up to even 128 independent receiver channels [3–6]. As the number of channels increases, however, problems of both data storage and processing complexity have emerged.

In order to achieve improved data processing efficiency in the large array systems, coil compression [7–10] was proposed. The concept of coil compression is to reduce the number of independent data streams before reconstruction by linearly combining raw data from multiple receiver channels into fewer virtual channels or selecting a small set of most important channels.

Coil compression can be applied in either hardware [10] or software. King et al. implemented an efficient channel reduction method through hardware by combining eight head coil elements into three channels using the idea of the noise covariance of the receiver arrays [10]. But coil compression in hardware is not always optimal because it does not take the spatial coil sensitivity variation or the received data into consideration.

Software coil compression is more flexible and accurate. Buehrer et al. demonstrated that array compression can be very effective by optimizing the signal-to-noise ratio (SNR) of the region of interest in the reconstructed image [7]. It requires exact coil sensitivities; therefore, this kind of coil compression is only fit for sensitivity-based PI reconstructions such as sensitivity encoding [1]. Doneva and Börnert proposed a different method to select the best subset coils by a ranking that quantifies the contribution of each coil to the final reconstructed image [8]. This method requires the coil sensitivities as well. Huang et al. developed a channel compression technique which is based on principal component analysis (PCA) [9]. This technique efficiently reduces the size of parallel imaging data acquired from multichannel coil arrays, thereby significantly reducing the level of computation cost without undermining the benefits of multichannel arrays. This method is very fast and it can work well with compressed sensing [11–14] and autocalibrating PI. Feng et al. proposed a method of reducing the computation burden in the* k*-domain PI, such as generalized autocalibrating partially parallel acquisitions (GRAPPA) [2], by utilizing localized sensitivity to calculate the cross-channel correlation for channel selection [15]. A new coil compression technique for Cartesian sampling was presented by Zhang et al. and it had less signal loss and more computation reduction through exploiting the spatially varying coil sensitivities in these nonsubsampled dimensions [16].

All the software coil compression methods above work well with Cartesian data acquisitions. However, coil compression applied in non-Cartesian sampling has been rarely reported. In this work, the geometric-decomposition coil compression (GCC), which was proposed by Zhang et al. with Cartesian sampling [16], was applied to accelerated imaging with radial acquisition for better compression.

In radial acquisition, the readout direction is always fully sampled and the variation of coil sensitivity along readout is not used for accelerating data acquisition. GCC can minimize the number of virtual coils by taking this spatially varying information into consideration. Both simulated MRI radial data from a 32-channel system and in vivo MRI radial data from a 52-channel system were tested. The experimental results show that the GCC method can achieve better reconstruction image quality than the conventional PCA approach in radial sampling in terms of SNR and normalized root mean squared error (NRMSE) values. Besides, the method is demonstrated using radial acquisition but is applicable to more general non-Cartesian *k*-space sampling trajectories.

## 2. Methods

The purpose of coil compression is to remove the redundancy which is caused by the correlation within receiver channels by decorrelating the data from different channels. It aims to reduce reconstruction time and data storage.

**Figure 1:**
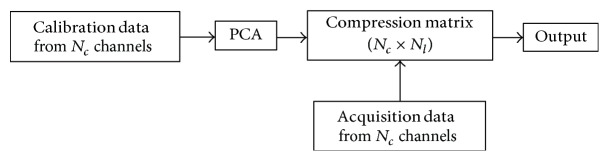
Traditional PCA-based coil compression method.

The traditional PCA-based coil compression method [9] is shown in Figure 1. It is also called single coil compression (SCC) because of the identical compression matrix. The calibration signal could be in *k*-space (before scan, autocalibration signal (ACS), and data with acceleration) or image space (low-resolution images, sensitivity profiles). The output of this system is the *k*-space data compressed into *N*_*l*_ channels.

Straightforward coil compression in *k*-space using the same compression matrix often requires a substantial number of virtual coils. This is due to data redundancy in the fully sampled direction. The following shows the steps of GCC method.

At each location *k* = [*k*_*x*_, *k*_*y*_] in *k*-space, *v*(*k*) = [*v*_1_(*k*), *v*_2_(*k*),…, *v*_*N*_(*k*)]^*T*^ represents data at this location from all the original coils. The compression matrices *A*_*x*_ in GCC can be separately calculated by singular value decomposition (SVD) at each *x* (*x* is the readout direction which is fully sampled; in this work, it represents the direction of *k*_*r*_ in radial MRI acquisitions) using the following steps [16]:

(1) Compute an inverse Fourier transform of the *k*-space multichannel data along the readout direction into [*x*, *k*_*y*_] coordinates.
(2) At each *x* location, construct a data matrix *X*_*x*_ in which each row consists of all the data *v*_*x*_(*k*_*y*_) from an individual original coil. Usually, ACS data are sufficient.
(3) Perform SVD of *X*_*x*_:
  (1) $$\begin{array}{c} X_{x}=U_{x}\underset{x}{\sum }{\left(\;V_{x}\;\right)}^{H}. \end{array}$$
Take the first *M* rows of (*U*_*x*_)^*H*^ to form an initial compression matrix *A*_*x*_^0^.
(4) Repeat Steps 2 and 3 to obtain compression matrices for all *x*.

In addition, using the solution *A*_*x*_^0^ obtained by SVD as the compression matrices is likely to introduce nonsmooth virtual coil sensitivities along the *x* direction. Therefore, alignment is necessary which is also clarified in Zhang's paper.

After the compression, GRAPPA [2] or iterative self-consistent parallel imaging reconstruction (SPIRiT) [17] is applied to reconstruct the final image. The GRAPPA reconstruction considers the parallel imaging reconstruction as a translation variant interpolation problem in *k*-space. GRAPPA is a self-calibrated channel-by-channel reconstruction method, which aims to reconstruct the individual channel images directly. In the GRAPPA algorithm, the nonacquired *k*-space value in the *i*th channel, at the position *r*, denoted as *x*_*i*_(*r*), is calculated by a linear combination of acquired neighboring *k*-space data from all channels. The linear combination weights, or calibration kernel, are obtained by calibration data from a fully acquired *k*-space region. The calibration chooses the set of weights that is the most consistent with the calibration data under the least-squares sense.

The SPIRiT is a general and efficient parallel imaging approach for arbitrary trajectories. The key in SPIRiT is to consider the consistency with not only the calibration but also the data acquisition. These constraints are formulated as sets of linear equations. Although the acquired *k*-space data may or may not be Cartesian, finally, the desired reconstruction is a complete Cartesian *k*-space grid for each of the channels. Consequently, we define the entire Cartesian *k*-space grid for all the channels as the unknown variables in the equations, making the formulation very general, which can be used for non-Cartesian sampling or so. More details can be seen in [17].

**Figure 2:**
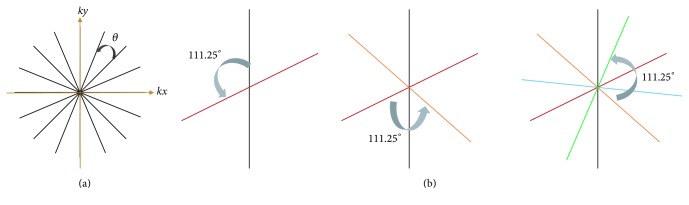
The *k*-space trajectory of (a) linear-angle and (b) golden-angle.

The GCC method was tested on a 32-channel dataset and a 52-channel dataset. The first dataset was fully sampled which was downloaded from the Internet (https://people.eecs.berkeley.edu/~mlustig/Software.html). According to Arunachalam et al., radial *k*-space data can be obtained by transforming the Cartesian *k*-space data into the Fourier domain and interpolating it into the desired linear-angle radial lines [18]. We obtained the radial data (192 readout points and 302 spokes) through this method. Imaging with the acceleration factors of 2, 3, 4, 5, and 6 was corresponding to the reduced dataset with 151, 101, 76, 60, and 50 spokes. The second dataset was acquired on a 3.0-tesla MR system (Siemens Healthcare, Erlangen, Germany) with golden-angle radial sampling using a 52-channel head coil array. The imaging parameters were as follows: pulse repetition time/echo time (TR/TE) = 4.08/2.03 ms, flip angle (FA) = 12°, field of view (FOV) = 220 × 220 mm^2^, slice thickness = 3 mm, number of readout points per spoke = 256, number of spokes = 400, and number of slices = 20. Four-hundred spokes were chosen as the reference, and the reduced dataset were 300 and 200 spokes, respectively. The 10th slice of each coil was tested using both SCC and GCC method. The third dataset was acquired on the same scanner with golden-angle radial acquisition using a body/spine coil array with 16 elements. Relevant imaging parameters included the following: TR/TE = 3.84/1.72 ms, FA = 12°, FOV = 385 × 385 mm^2^, slice thickness = 3 mm, number of readout points per spoke = 256, number of spokes = 420, and number of slices = 10. Frequency-selective fat suppression was used for both acquisitions. Gradient-delay errors were corrected before image reconstruction. The linear-angle radial trajectory [19, 20] and the golden-angle radial trajectory [21, 22] were shown in Figures 2(a) and 2(b), respectively. Linear-angle sampling has a constant azimuthal spacing of *θ* = *π*/spokes. Golden-angle sampling has a constant azimuthal spacing of 111.25°. Radial sampling usually has undersampling tolerance and motion robustness.

The difference map, NRMSE, and SNR were used to evaluate the quality of the reconstructed images. The square root of sum-of-square (SSOS) image from all the original coils without acceleration was used as the reference. The NRMSE and SNR were calculated using the formula as follows:

(2) NRMSE=∑Irecon−Iref2∑Iref2,SNR=10×log10⁡∑Irecon2∑Irecon−Iref2,

where *I*_ref_ is the reference image and *I*_recon_ is the reconstructed image.

## 3. Results

**Figure 3:**
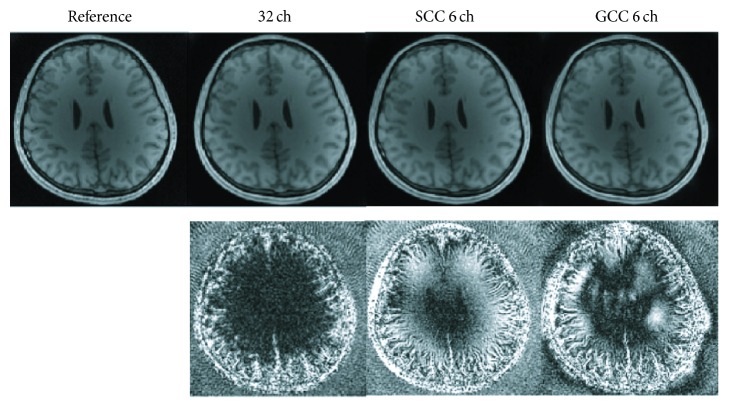
Comparison of SCC and GCC for simulation results with acceleration factor of 2 in angle direction. SSOS images are compared. The original image in the first column is used as the reference. The compression results of SCC and GCC with 6 virtual coils are shown in the third and fourth column, respectively. Difference maps (10x in SCC and GCC) are shown in the second row. The *k*-space trajectory is in linear-angle pattern. (The images and the difference maps are displayed in the same gray scale range, resp.; and Figures 5, 6, 8, and 10 are as well.)

The number of virtual coils was empirically set to be 6 for both SCC and GCC in the first dataset. The *k*-space trajectory was in linear-angle pattern. The experimental results with acceleration factor of 2 in angle direction of the first dataset are shown in Figure 3. Compared with the reference image, the SCC compression result shows more signal loss while the GCC looks similar to the reference image. The 10x difference map between SCC image and the reference image further indicates large compression error owing to the nonoptimal coil compression method. However, the compression error in GCC image is smaller. The NRMSE values are 0.0632 for SCC and 0.0577 for GCC. The SNR values are 15.8 dB for SCC and 17.3 dB for GCC. The experimental results show that GCC has much better compression performance than SCC for the same number of virtual coils.

**Figure 4:**
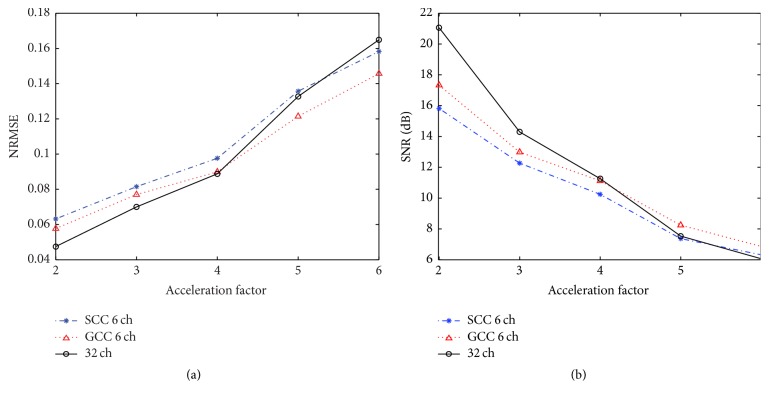
Plot of (a) NRMSE and (b) SNR at different acceleration factors (2 to 6) of data from 32 original coils, 6 virtual coils in SCC, and 6 virtual coils in GCC. SSOS image of data from 32 original coils with full sampling is used as the reference.

The same experiment was done at different acceleration factors from 2 to 6. The NRMSE and SNR with different acceleration factors are shown in Figures 4(a) and 4(b), respectively. This quantitative analysis further confirms the following observations: (a) the NRMSE and SNR values of the reconstructed images with 32 original coils, 6 virtual coils in SCC, and 6 virtual coils in GCC are always close; (b) when the acceleration factor is low (2 or 3), data from 32 original coils generate the least errors and the highest SNR; (c) when the acceleration factor is high (5 or 6), data from 6 virtual coils in GCC generate the least errors and the highest SNR. These results imply that data from 32 original coils has better PI performance at acceleration factor of 2 and 3. However, it is interesting that data from 6 virtual coils in GCC generate better results than those from 32 original coils at acceleration factors 5 and 6.

**Figure 5:**
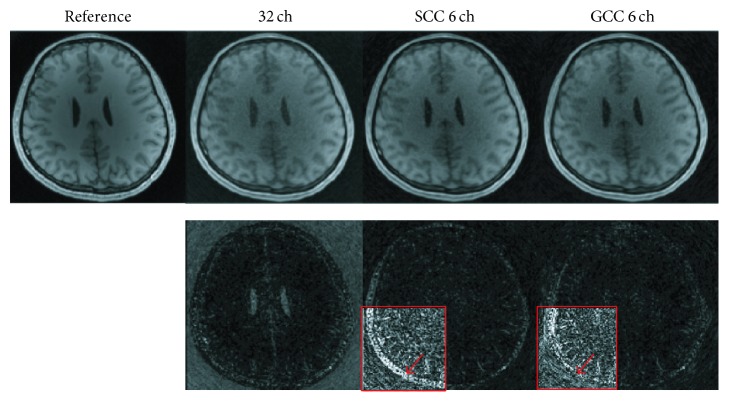
Results of denoising from noisy dataset of 32 original coils, 6 virtual coils in SCC, and 6 virtual coils in GCC. The SSOS image of the noiseless dataset is used as the reference. Difference maps (4x enlarged view in red box) are shown in the second row.

The SSOS images of all the reconstructions in the denoising experiment are shown in Figure 5. We added white Gaussian noise (*δ* = 0.005 for each coil). The number of virtual coils (both SCC and GCC) was chosen to be 6 and the acceleration factor was 2. The denoising effect of SCC and GCC can be seen as the background noise is reduced after coil compression. The results of SCC and GCC with added noise are also compared in the red box. GCC has better noise suppression than SCC for the same number of virtual coils at the same acceleration factor.

**Figure 6:**
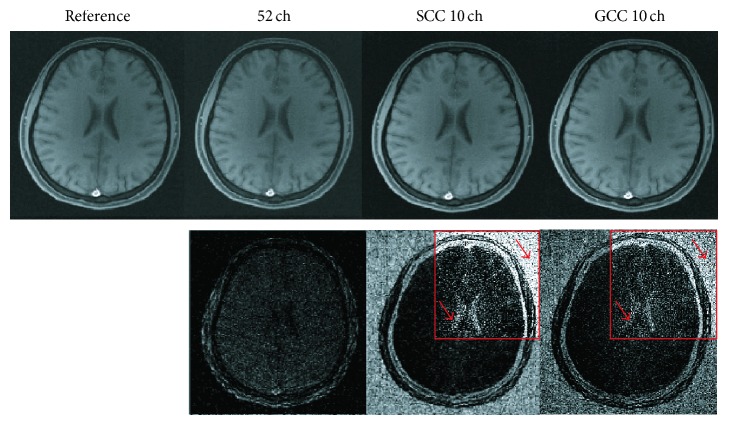
Comparison of SCC and GCC results for in vivo undersampled 300 spokes' data. SSOS images are compared. The original image in the first column is used as the reference. The compression results of SCC and GCC with 10 virtual coils are shown in the third and fourth column, respectively. Difference maps (4x enlarged view in red box) are shown in the second row. The *k*-space trajectory is in golden-angle pattern. The reconstructions of the undersampled data are performed by SPIRiT.

The radial data used above were all acquired in the linear-angle pattern. Another widely used pattern is the golden-angle radial sampling. Therefore, we use this kind of radial data to further evaluate the effect of SCC and GCC. The second dataset from 52 channels was used in this experiment. The number of virtual coils was empirically set to be 10 for both SCC and GCC. 300 spokes were used for undersampling. The SSOS image of the data from 52 original coils without acceleration of 400 spokes was used as the reference. The results are shown in Figure 6. Compared with the reference image, the SSOS image of the undersampled data from 52 coils without compression has suffered more noise which can be seen from the noisy background. And its NRMSE value is 0.0453 and SNR value is 26.1 dB. The 4x difference map in the red box of Figure 6 between SCC image and the reference image further indicates large compression error due to the noisier background, while the compression error in GCC image is smaller. The NRMSE values are 0.0796 for SCC and 0.0686 for GCC. The SNR values are 21.4 dB for SCC and 22.6 dB for GCC. The in vivo data results further show that GCC has better compression performance than SCC for the same number of virtual coils.

**Figure 7:**
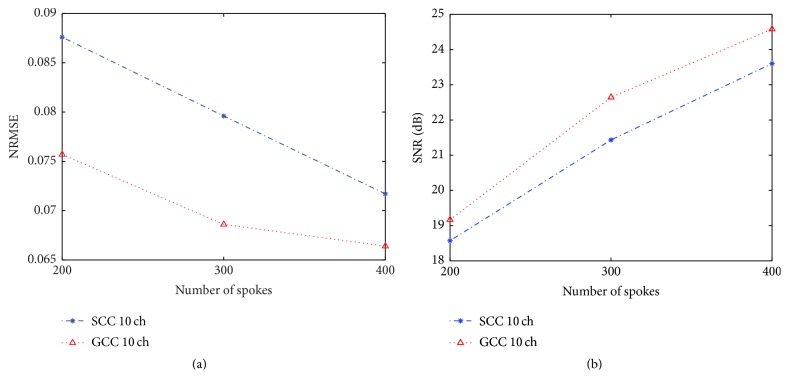
Comparison of (a) NRMSE and (b) SNR at different numbers of spokes from 200 to 400 of data from 10 virtual coils in SCC and 10 virtual coils in GCC. SSOS image of data from 52 original coils with 400 spokes is used as the reference.

Figures 7(a) and 7(b) show the NRMSE and SNR values at different numbers of spokes from 200 to 400 in SCC and GCC, respectively. From the curve below, it is found that GCC shows lower NRMSE and higher SNR than SCC at all these different numbers of spokes. To achieve similar NRMSE and SNR as in GCC, SCC should be applied to smaller acceleration factor. GCC shows its obvious strength in coil compression and it also has the advantage of great undersampling robustness. In addition, it is obvious that the result from 52 original coils without any compression has become worse with the reduction in the number of spokes. When the number of spokes reduces to 200, the NRMSE of SSOS image from 52 coils increases to 0.0925 and SNR decreases to 17.7 dB. Both the image quality of SCC (NRMSE = 0.0876, SNR = 18.6 dB) and the image quality of GCC (NRMSE = 0.0757, SNR = 19.2 dB) are better than the result of 52 coils.

**Figure 8:**
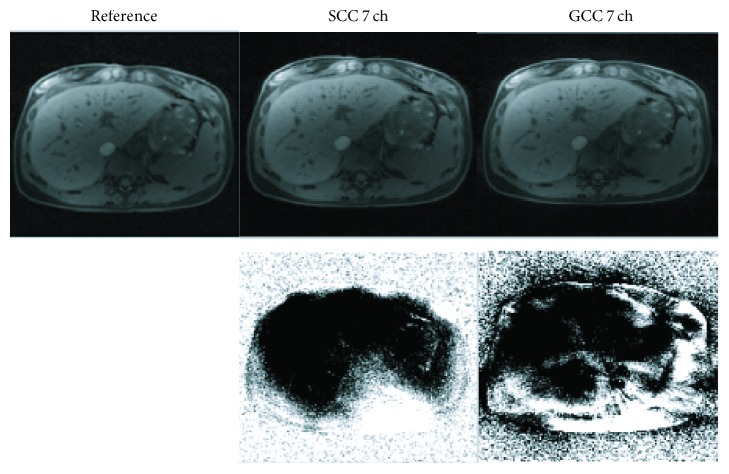
Comparison of SCC and GCC with 7 virtual coils for in vivo abdomen data results in fully sampled golden-angle scheme. SSOS image from 16 original coils is used as the reference. Difference maps are shown in the second row.

Figure 8 shows the results of SCC and GCC of the in vivo fully sampled 16-channel abdomen dataset. SSOS image from 16 original coils is used as the reference. Compared with the reference image, GCC has less compression loss with 7 virtual coils, while SCC suffers from larger compression loss. The NRMSE and SNR values of SCC and GCC are 0.0374 and 32.9 dB and 0.0363 and 33.2 dB, respectively.

## 4. Discussion

In this work, we have studied GCC in non-Cartesian parallel imaging with radial acquisition. Figures 3, 4, 6, and 7 show that the reduction of channels in SCC and GCC does not degrade the final image quality in terms of NRMSE and SNR. In addition, GCC has less image detail loss than SCC for the same number of virtual coils at the same acceleration factor. It is because that GCC takes the advantage of the coil sensitivity variation in the readout direction which is not used for acceleration in data acquisition. The acquired data can be spatially separated in the readout direction. Thus GCC can effectively minimize the number of virtual coils at each location of readout direction to reduce the reconstruction time. Figure 8 indicates that GCC can also effectively compress abdomen dataset. Thus, GCC can also be applied to effectively compress time-series data from large arrays, for example, in many dynamic imaging experiments.

We have chosen 6 virtual coils for the first 32-channel dataset, 10 for the second 52-channel dataset, and 7 for the third 16-channel dataset. In fact, the number of effective virtual coils can be determined by thresholding the singular values in SVD at the third step. In addition, the NRMSE and SNR can also be useful reference indexes to choose the appropriate number of virtual coils.

**Figure 9:**
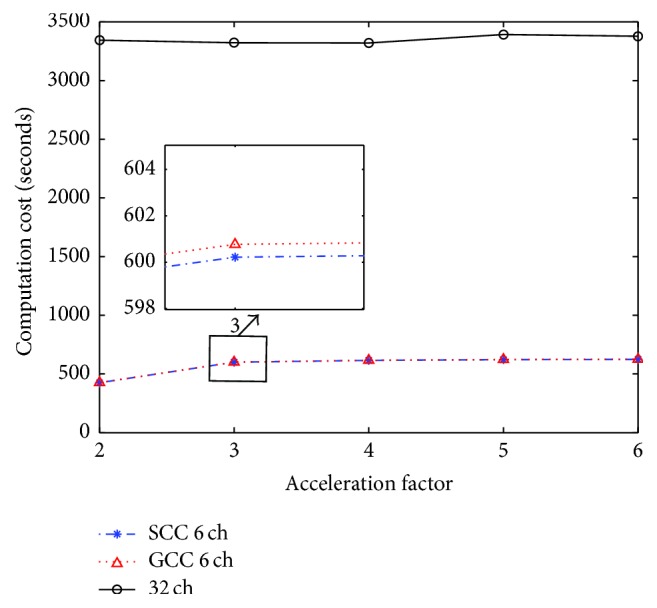
Computation cost of SCC and GCC for the first dataset at different acceleration factors.

The main purpose of coil compression is to reduce data storage and the reconstruction time. For parallel imaging reconstruction algorithms, such as GRAPPA and SPIRiT, the introduction of SCC and GCC will significantly accelerate the speed of image reconstruction. Taking the first dataset as an instance, computation cost of SCC and GCC at different acceleration factors is shown in Figure 9. SCC and GCC are about six times less than the reconstruction without compression from all the original coils. The reason for this advantage is that both SCC and GCC use fewer channels than the conventional reconstruction scheme. SCC is slightly faster than GCC because GCC has an alignment procedure.

While adding noise into the original coils, SCC and GCC show more noise tolerance than the conventional noncompression method, as shown in Figure 5. The denoising effect of coil compression can be considered as the selectively discarded virtual coils do not contain significant signal and are dominated by the added noise. Besides, GCC has better noise suppression than SCC for the same virtual coils at the same acceleration factor. This is because GCC can remove more noise than SCC by taking the fully sampled direction into consideration.

**Figure 10:**
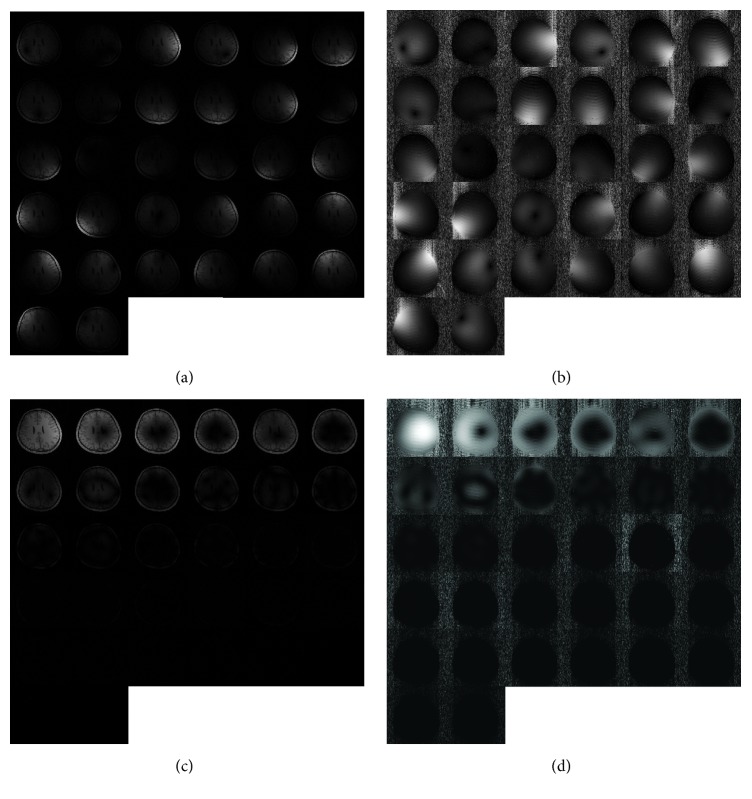
Images and sensitivity maps of 32 coils before ((a) and (b)) and after ((c) and (d)) coil compression.

However, it is interesting that data from all the original coils generate more signal loss than those from virtual coils in SCC and GCC at a high acceleration factor, such as 5 and 6 in the first dataset and spokes of 200 in the second dataset. This can be interpreted as the different noise levels in the data before and after compression.  Figure 10 shows that the coils we selectively discarded have hardly any signal. The noise in these coils is comparable to signal levels in them. Data from 6 virtual coils in SCC and GCC have less noise and fewer signals when compared to the data from 32 original coils. When the acceleration factor is low, the *g*-factor is low. For example, when the acceleration factor is 2, the *g*-factors of 32 ch, SCC 6 ch, and GCC 6 ch are 1.07, 1.18, and 1.14, respectively. Therefore, the reconstruction errors are dominated by the lost signal in the discarded coils. So it can be concluded that the results of data from 32 original coils are better. When the acceleration factor goes higher, for example, when the acceleration factor is 6, the *g*-factors of 32 ch, SCC 6 ch, and GCC 6 ch are 3.15, 17.96, and 10.91, respectively. The noise is dramatically increased and is more dominant in the reconstruction errors. When the coils reduce, the SNR increases as the noise suppression is more than the decrease due to the signal loss. Therefore, the results of data from 6 virtual coils in SCC and GCC are better than those from 32 original coils. More noise will be introduced when the data is much more undersampled. SCC and GCC have better noise robustness owing to the fact that they can selectively discard the useless virtual coils.

In this work, the GCC method was first applied to radial parallel imaging to accelerate image reconstruction. GCC was also tested with different acceleration factors in angle direction, which further indicated its validity and robustness in terms of NRMSE and SNR. Moreover, this feature is particularly obvious in highly undersampled data.

## 5. Conclusion

We have extended the coil compression method GCC to radial acquisitions in this work, and both linear-angle and golden-angle patterns were studied in this paper. The image quality of reconstructions using virtual coils in GCC is always comparable to that of reconstructions using all the original coils at all the tested acceleration factors. In addition, GCC can achieve better compression result than SCC for the same number of virtual coils.
